# GLP-1 Receptor Agonist Use in Patients Undergoing Distal Radius Fracture Surgery: A Retrospective Review of Perioperative Considerations

**DOI:** 10.7759/cureus.89183

**Published:** 2025-07-31

**Authors:** Mohammad Khak, Jeremiah A Adams, Muhammad Hozien, Asif M Ilyas

**Affiliations:** 1 Orthopaedic Surgery, Rothman Orthopaedic Institute, Philadelphia, USA; 2 Orthopedic Surgery, Rothman Orthopaedic Institute at Thomas Jefferson University, Philadelphia, USA; 3 Thomas Jefferson University Hospital, Sidney Kimmel Medical College, Philadelphia, USA; 4 Orthopaedic Surgery, Drexel University College of Medicine, Philadelphia, USA; 5 Orthopaedics, Rothman Orthopaedic Institute at Thomas Jefferson University, Philadelphia, USA

**Keywords:** distal radius fracture, glp-1 receptor agonists, orthopaedic anesthesia, perioperative complications, semaglutide

## Abstract

Introduction: Glucagon-like peptide-1 receptor agonists (GLP-1 RAs) are increasingly prescribed for glycemic control and weight loss, but their effects on surgical outcomes remain incompletely understood. Delayed gastric emptying and associated perioperative risks have prompted updated anesthetic guidelines, although evidence in orthopaedic or hand surgery remains limited. This study aims to evaluate the prevalence of GLP-1 RA use and its perioperative implications among patients undergoing surgical fixation for distal radius fractures (DRFs).

Methods: A retrospective review of 4,811 adult patients undergoing operative treatment for DRFs between 2019 and 2024 was conducted. GLP-1 RA use prior to surgery was identified through medication reconciliation. Demographics, comorbidities, medication type, and postoperative complications, including nausea, vomiting, abdominal pain, aspiration, emergency visits, and hospital readmissions, were assessed.

Results: GLP-1 RA use was observed in 46 patients (1.0%) over a six-year period, with prevalence rising from 0.4% to 0.5% annually between 2019 and 2022 (three to four patients each year) to 1.0% in 2023 (eight out of 808 patients) and to 2.9% in 2024 (23 out of 781 patients). Semaglutide was the most commonly used agent in the cohort. Only one GLP-1 user (2.2%, one out of 46) experienced postoperative nausea. There were no cases of aspiration or serious complications documented.

Conclusion: GLP-1 RA use among orthopaedic trauma patients is increasing, reflecting broader prescribing trends. In this cohort, perioperative complications were rare, although underreporting may be a factor. The retrospective nature of the study and the relatively small number of GLP-1 users limit the generalizability of the findings. Nonetheless, orthopaedic surgeons should be aware of potential anesthetic considerations, evolving guidelines, and the importance of coordinated perioperative planning as GLP-1 use expands.

## Introduction

With rates of obesity, type 2 diabetes, and metabolic syndrome continuing to rise in the United States, the development of safe and effective pharmacologic agents has become a major focus within the pharmaceutical industry [1,2]. Among the most widely adopted therapies in recent years are glucagon-like peptide-1 receptor agonists (GLP-1 RAs), including semaglutide, dulaglutide, liraglutide, and tirzepatide [3]. These medications have gained increasing public attention due to their dual role in glycemic control and weight loss [4-6].

GLP-1 RAs exert their metabolic effects by enhancing endogenous insulin secretion, suppressing glucagon release, delaying gastric emptying, and promoting satiety [4,7]. While they have provided significant benefits in managing obesity and type 2 diabetes, their pharmacologic profile has important implications for surgical care. Improved glycemic control and weight reduction may allow previously ineligible patients to meet preoperative safety thresholds, potentially lowering surgical risks and postoperative complications [5, 8].

However, the delayed gastric emptying induced by GLP-1 RAs has raised concerns in perioperative medicine. Specifically, the risk of residual gastric contents at the time of anesthesia induction may increase the likelihood of gastrointestinal symptoms such as nausea, vomiting, and abdominal pain, and more critically, the risk of pulmonary aspiration [9-11]. These concerns have led to the publication of new fasting and medication-holding guidelines by anesthesia societies, but clear protocols are lacking in orthopaedic and hand surgical settings [6,12].

The primary aim of this study was to report the incidence of perioperative GLP-1 RA use among patients undergoing surgical treatment for distal radius fractures (DRFs) at a high-volume academic orthopaedic practice. Secondary objectives were to describe associated perioperative complications and to propose practical considerations for surgeons managing patients who are prescribed GLP-1 RAs.

## Materials and methods

This study was approved by the Rothman Institute / Thomas Jefferson University Institutional Review Board (IRB) (Control #13D.432). As a retrospective review of de-identified data, patient consent was waived. A retrospective cohort study of adult patients who underwent surgical fixation of DRFs at a high-volume academic orthopaedic hand and wrist practice was conducted between January 1, 2019, and December 31, 2024. Surgical procedures included both open reduction and internal fixation (ORIF identified via CPT codes 25607, 25608, and 25609) and closed reduction and internal fixation (CRIF identified via CPT code 25606). Patients who underwent surgery under general or regional anesthesia were included, while those operated under local anesthesia were excluded.

A query of institutional databases identified 4,724 patients who underwent operative treatment for DRFs during the study period. Available pharmacy records were reviewed to determine the use of GLP-1 RAs. The search for GLP-1 RAs included dulaglutide (Trulicity), exenatide (Byetta, Bydureon), liraglutide (Victoza, Saxenda), lixisenatide (Adlyxin), semaglutide (Ozempic, Rybelsus, Wegovy), and tirzepatide (Mounjaro, Zepbound). Patients were categorized into two groups, GLP-1 RA users or non-users, based on documented GLP-1 RA use prior to surgery. Patients were categorized as perioperative GLP-1 RA users if the pharmacy “fill date” and “days supply” data could be used to support patient access to medication no greater than 14 days preoperatively. At the surgical centers where the patients were treated, the standard perioperative protocol recommended withholding GLP-1 receptor agonists for at least seven days prior to surgery for weekly injectable formulations and for at least one day prior to surgery for daily oral formulations, in accordance with institutional anesthesiology guidelines.

Electronic health records were reviewed for all patients in the GLP-1 RA group. Extracted data included demographic information (age, gender, and body mass index), comorbidities (type 2 diabetes mellitus, hypertension, hyperlipidemia, and cardiovascular disease), and confirmation of preoperative (within 14 days) GLP-1 RA use. Medication type and the timing of the most recent GLP-1 RA dose were also recorded. Postoperative outcomes included documentation of complications within 15 days of surgery, such as nausea, vomiting, abdominal pain, and pulmonary aspiration. Additional postoperative follow-up data included records of nursing follow-up calls, emergency department visits, and unplanned hospital admissions.

The primary outcome was the incidence of GLP-1 RA use in patients undergoing DRF surgery. Secondary outcomes included the rate of perioperative complications and descriptive characteristics of GLP-1 RA users. Descriptive statistics were used to summarize patient characteristics. Continuous variables were reported as mean ± standard deviation and compared using Student’s t-test or Mann-Whitney U test as appropriate. Categorical variables were presented as counts and percentages and compared using chi-square or Fisher’s exact test. The annual prevalence of GLP-1 RA use was calculated and plotted over the study period.

## Results

**Figure 1:**
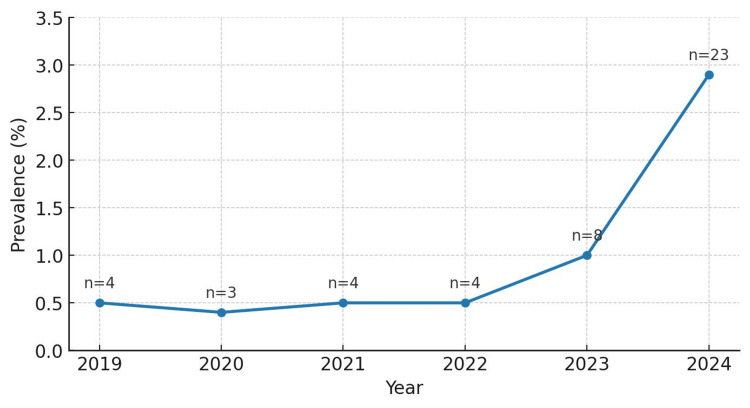
Prevalence of GLP-1 receptor agonist use in operative distal radius fracture patients over the study period (2019-2024)

Between January 2019 and December 2024, a total of 4,811 wrists in 4,724 adult patients underwent surgical fixation (ORIF or CRIF) for distal radius fractures under general or regional anesthesia. Over the six-year study period, 46 out of 4,811 wrists (1.0%) undergoing distal radius fracture surgery were GLP-1 users. However, GLP-1 use increased sharply in recent years, rising from 0.4 to 0.5% annually between 2019 and 2022 (three to four patients each year) to 1.0% in 2023 (eight out of 808 patients) and to 2.9% in 2024 (23 out of 781 patients) (Figure 1, Table 1).

**Table 1:** Annual prevalence of GLP-1 receptor agonist use among patients undergoing distal radius fracture (DRF) surgery from 2019 to 2024.

Year	Total DRF surgeries	GLP-1 users	Prevalence (%)
2019	801	4	0.5
2020	753	3	0.4
2021	828	4	0.5
2022	840	4	0.5
2023	808	8	1.0
2024	781	23	2.9
Total	4811	46	1.0

The mean age of GLP-1 RA users was 64.0 ± 9.5 years, compared to 59.7 ±15.8 years in non-users. A higher percentage of GLP-1 RA users were female. Patients on GLP-1 RAs had a significantly higher mean BMI compared to non-users (33.9 ± 7.6 vs. 26.9 ± 5.8, p-value < 0.05). Type 2 diabetes, hypertension, hyperlipidemia, and cardiovascular disease were more common in the GLP-1 RA group (Table 2).

**Table 2:** Baseline characteristics of patients undergoing distal radius fracture surgery, stratified by GLP-1 receptor agonist (GLP-1 RA) use. Continuous variables are presented as mean ± standard deviation (range), and categorical variables as counts with percentages.

Variable	GLP-1 RA group (n = 46)	Non–GLP-1 RA group (n = 4765)	p-value
Age (years), mean ± SD; (range)	64.0 ± 9.5; (39 - 89)	59.7 ± 15.8; (18 - 100)	<0.05
Female, n (%)	37 (80.4%)	3654 (76.7%)	NS
BMI, mean ± SD	33.9 ± 7.6	26.9 ± 5.8	<0.05
Type 2 diabetes, n (%)	13 (28.3%)	167 (3.5%)	<0.05
Hypertension, n (%)	22 (47.8%)	1041 (21.8%)	<0.05
Cardiovascular disease, n (%)	5 (10.9%)	312 (6.5%)	NS
Hyperlipidemia, n (%)	18 (39.1%)	974 (20.4%)	<0.05

**Figure 2:**
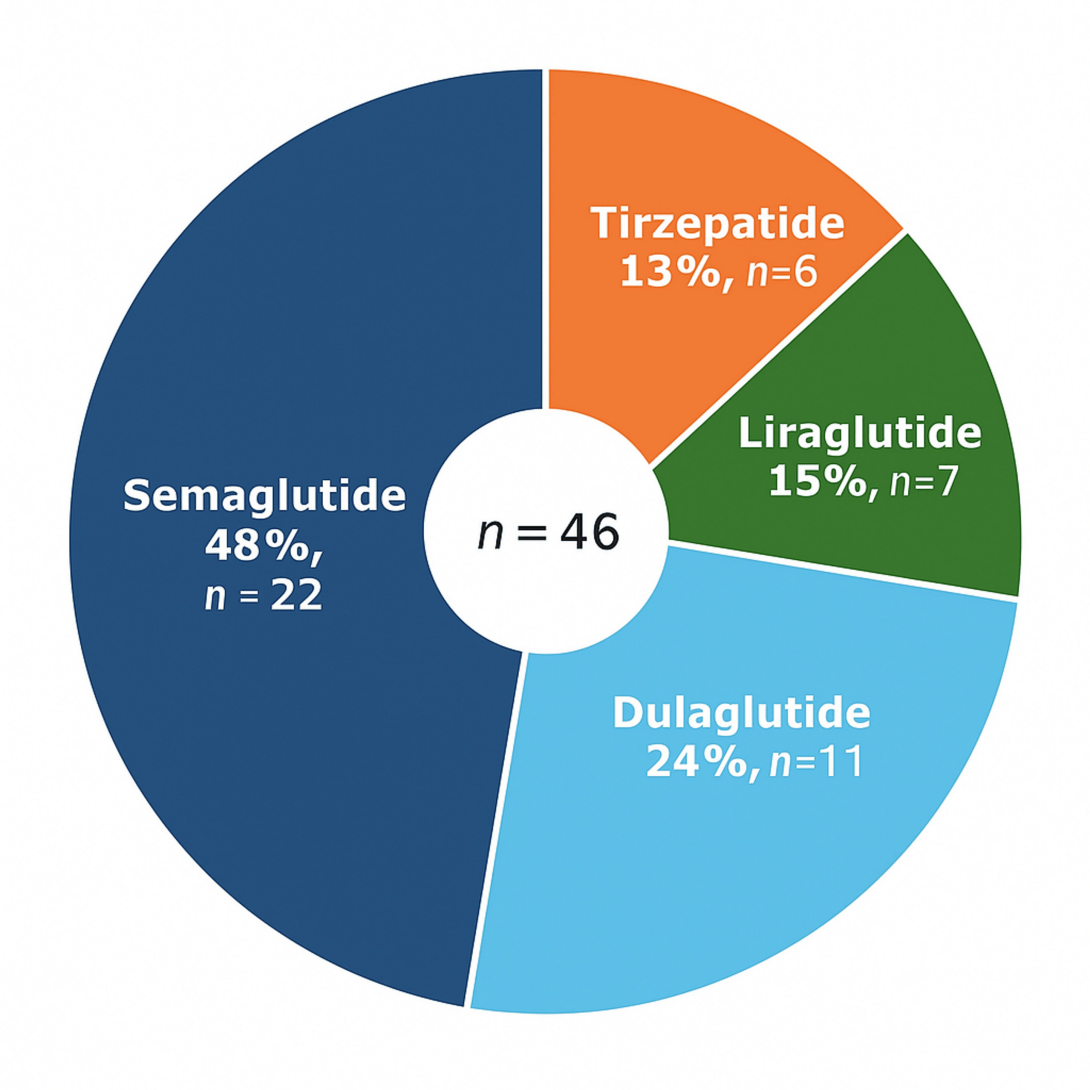
Distribution of GLP-1 receptor agonist types among users in the study cohort.

Among GLP-1 RA users, the most commonly used medications were semaglutide (22 of 46, 48%), followed by dulaglutide (11 of 46, 24%) (Figure 2). Among all patients, the most common procedures were ORIF (4,775 of 4,811, 99.25%).

Only one GLP-1 user (2.2%, one out of 46) experienced documented postoperative nausea and vomiting, which was reported within 24 hours after surgery and was managed with a single dose of intravenous ondansetron. There were no cases of abdominal pain, pulmonary aspiration, emergency room visits, or hospital readmissions within 15 days of surgery.

## Discussion

In this study, an increase in GLP-1 receptor agonist use was observed among adult patients undergoing surgical fixation for distal radius fractures over a six-year period. While the overall prevalence of GLP-1 use remained low at 46 of 4,811 patients (1.0%) across all patients treated between 2019 and 2024, a sharp rise was evident in the latter years, reaching 23 of 781 patients (2.9%) in 2024. This trend mirrors broader prescribing patterns nationwide, where GLP-1 receptor agonists have seen expanded use not only for glycemic control in type 2 diabetes mellitus but also increasingly for weight management in obese and overweight populations. Among the 46 patients on GLP-1 RAs, only one reported postoperative nausea and vomiting, and no cases of pulmonary aspiration, abdominal pain, or emergency department visits were documented. These findings suggest that while GLP-1 RA use is increasingly common in orthopaedic and hand surgical patients, the incidence of immediate perioperative complications in this setting appears to be low.

The low complication rate observed in this cohort contrasts with findings from clinical trials of GLP-1 RAs in non-operative populations, which consistently report nausea, vomiting, and other gastrointestinal symptoms as frequent adverse effects. A systematic analysis found that these symptoms were common across nearly all trials of GLP-1 RAs, particularly during the dose-escalation phase [13]. Similarly, delayed gastric emptying and nausea are described as central features of GLP-1 RA pharmacology, which may explain the heightened concern for their use in surgical patients [14].

This study’s findings align with the notion that complications such as nausea and vomiting may be underreported in outpatient orthopaedic and hand surgery settings. Unlike inpatient procedures, such as total hip arthroplasty, where postoperative monitoring is continuous, hand surgeries are typically performed on an outpatient basis, and minor adverse effects may go unrecognized or unreported. In a recent study of GLP-1 RA use in total hip arthroplasty, a higher incidence of nausea and vomiting among GLP-1 users was reported, likely due to more consistent in-hospital documentation [15]. In contrast, patients undergoing hand surgery are often discharged shortly after the procedure and may attribute symptoms such as nausea to their routine medication rather than report them to their care team.

Concerns about residual gastric content and delayed gastric emptying have led to evolving recommendations around GLP-1 RA management in the perioperative period. A study showed that patients taking semaglutide had significantly higher residual gastric contents during upper endoscopy compared to those who had withheld the medication, directly implicating GLP-1 RAs in aspiration risk [16]. These findings have been echoed by recent multisociety guidelines issued by the American Society of Anesthesiologists and collaborating organizations, which recommend holding GLP-1 RAs prior to surgery, especially in patients receiving general anesthesia or those at higher risk of aspiration [6].

Other emerging considerations for GLP-1 RA users include the potential impact of reduced dietary intake on wound healing. Some authors raised concerns that patients on these medications tend to consume fewer calories, including protein, which could impair postoperative recovery, particularly in surgeries requiring soft-tissue repair [17]. While we did not assess wound healing in this study, future investigations may benefit from examining nutritional markers and longer-term surgical outcomes in this population.

Importantly, this study’s findings reflect real-world practice in a high-volume outpatient orthopaedic and hand surgical setting. While the absence of significant complications in our GLP-1 RA cohort is reassuring, it also underscores the need for formalized preoperative screening questions, multidisciplinary coordination, and thoughtful decision-making around anesthesia modality and medication management. As the prevalence of GLP-1 RA use continues to rise, particularly with expanded indications for weight loss, surgeons and anesthesiologists must remain vigilant and adaptable in their approach.

This study has several limitations that warrant consideration. First, as a retrospective review, it is subject to the inherent biases of chart-based data collection, including variability in documentation and incomplete reporting of symptoms such as nausea or vomiting. Given that the majority of distal radius fracture surgeries were performed in the outpatient setting, postoperative adverse effects may have been underreported, particularly if patients did not seek medical attention or did not mention symptoms during follow-up calls. Additionally, while operative time and anesthesia type were available for the GLP-1 RA group, comparable data were not accessible for non-users, limiting our ability to perform a full comparative analysis. The sample size of GLP-1 RA users was relatively small, which may limit the generalizability of our findings and preclude definitive conclusions about the safety profile of these medications in this setting. Finally, we were unable to account for variability in medication dosing schedules or timing of the last dose relative to surgery in a standardized fashion. Moreover, it is unclear if the findings would have been different if surgery had not been held for at least a week when a patient was taking GLP-1 RA, per anesthesia protocol.

## Conclusions

In this retrospective review, GLP-1 RA use was observed in 46 of 4,811 patients (1.0%) between 2019 and 2024, with a sharp rise in the latter years, reaching 23 of 781 patients (2.9%) in 2024. Only one GLP-1 RA user (one of 46, 2.2%) experienced postoperative nausea, and no cases of aspiration or other serious perioperative complications were documented. While these findings are reassuring, they should be interpreted in the context of limited postoperative observation and a small sample size. As clinical adoption of GLP-1 RAs continues to expand, surgeons and perioperative teams should remain informed of emerging guidelines, coordinate closely with anesthesia providers, and consider thoughtful preoperative screening for medication use, anesthesia planning, and nutritional status. Further prospective, multicenter studies are needed to establish standardized protocols for perioperative GLP-1 RA management in orthopaedic and hand surgery.
